# 630. Sustained reduction of meropenem use in a tertiary-care pediatric service in Medellín, Colombia

**DOI:** 10.1093/ofid/ofae631.195

**Published:** 2025-01-29

**Authors:** Alejandro Diaz Diaz, Adriana M Echavarria-Gil, Carolina Jimenez, Juan Gonzalo Mesa-Monsalve

**Affiliations:** Hospital General de Medellin, Medellin, Antioquia, Colombia; Hospital General de Medellin, Medellin, Antioquia, Colombia; Hospital General de Medellin, Medellin, Antioquia, Colombia; Hospital General de Medellin/Clínica Las Américas Auna, Envigado, Antioquia, Colombia

## Abstract

**Background:**

Carbapenems are the widest spectrum antibiotics, categorized as a Watch group in the AWaRe classification tool by the World Health Organization, which recommends limiting its use only to certain specific infections. In settings where circulation of multidrug resistant Gram-negative organisms is common, overuse of carbapenems might occur which leads to additional costs and harm. Antibiotic stewardship programs (ASP) have demonstrated impact on antibiotic use in the hospital setting. We describe the impact of the ASP in meropenem use in children at a large public hospital.

**Figure:**
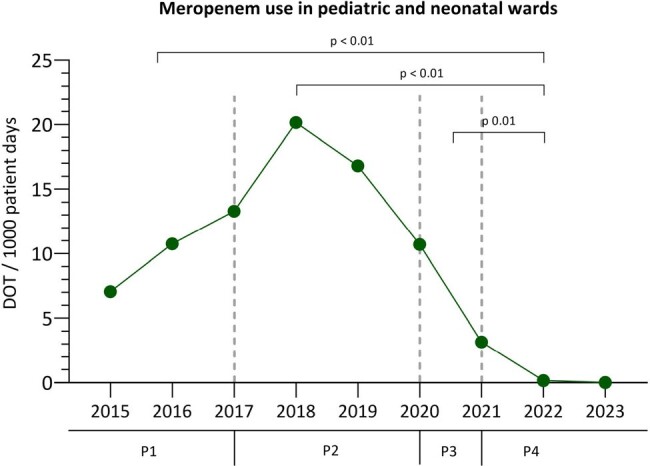

P: period; DOT: days of therapy

**Methods:**

Single-center (largest regional public hospital: 450-bed (110 pediatric (55 NICU + neonatal ward – 55 PICU + pediatric ward), retrospective observational study. ASP was established in late 2014. Initial strategies included weekly hand-shake antibiotic rounds led by a pediatric infectious disease physician. Multimodal ASP was implemented hospital-wide in 2016. During 2017 to 2019 a surge in resistant *Enterobacter* spp invasive infections occured in the NICU, which affected antibiotic treatment temporarily. Hence, we divided Observation period from 2015 – 2023 in four periods (P): P1 2015-2017: ASP establishment; P2 2017-2019: increase in invasive infections caused by *Enterobacter* spp resistant to cephalosporins; P3 2020: COVID-19 pandemic; P4 2021-2023: post-pandemic (figure 1). Meropenem consumption was measured with days of therapy (DOT). We used descriptive statistics for analysis.

**Figure:**
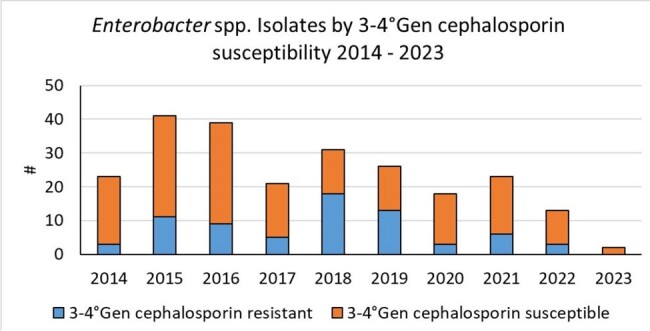

**Results:**

Meropenem use increased significantly during P1 (DOT 9.05 [5.8-16] and P2 (DOT 19.5 [15.2-24.3]; p< 0.01). Reinforcement of infection control measures and updated antibiotic guidelines with restricted meropenem indications with pre-authorization by the ID team led to sustained decrease of meropenem use after P2. This trend was not affected by COVID-19 pandemic (P3 DOT 10.7 [6.8-12-8] and P4 DOT 1.1 [0-3]; p ≤ 0.01). The number of highly resistant Enterobacter isolates also decreased over time while ESBL rates remained unchanged (Figure 2 & 3).

**Figure:**
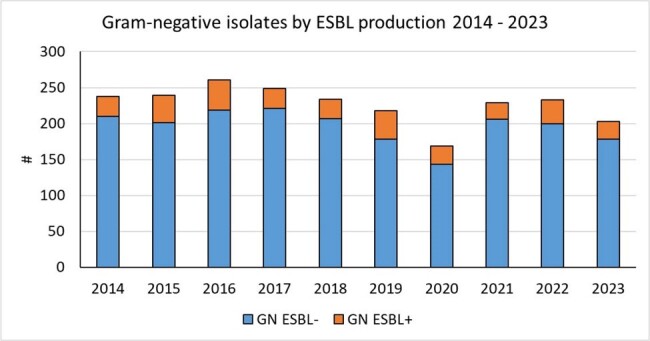

ESBL: Extended spectrum beta-lactamase

**Conclusion:**

ASP and infection control policies led to a significant and sustained decrease in meropenem use in our pediatric service. Leadership, administrative support, teamwork, and continued education are important to achieve goals in antibiotic consumption.

**Disclosures:**

**All Authors**: No reported disclosures

